# From superhydrophilicity to superhydrophobicity: high-resolution neutron imaging and modeling of water imbibition through porous surfaces treated with engineered nano-coatings

**DOI:** 10.1038/s41598-023-38324-1

**Published:** 2023-07-10

**Authors:** Filip Zemajtis, Abul Borkot Md Rafiqul Hasan, Okan Yetik, Pavel Trtik, Krishna M. Pillai, Konstantin Sobolev

**Affiliations:** 1 Department of Materials Science and Engineering, University of Wisconsin-Milwaukee, Milwaukee, WI 53211 USA; 2 Department of Mechanical Engineering, University of Wisconsin-Milwaukee, Milwaukee, WI 53211 USA; 3 Laboratory for Neutron Scattering & Imaging, Paul Scherrer Institut, CH-5232 Villigen PSI, Switzerland; 4 Laboratory for Nuclear Materials, Paul Scherrer Institut, CH-5232 Villigen PSI, Switzerland; 5 Department of Civil & Environmental Engineering, University of Wisconsin-Milwaukee, Milwaukee, WI 53211 USA

**Keywords:** Materials science, Nanoscience and technology

## Abstract

This paper reports on a superhydrophilic to superhydrophobic transformation of TiO_2_ nanoparticles doped zinc phosphate coating systems when a hydrophobic agent is applied. The objective of the reported research was to demonstrate the feasibility of a neutron imaging technique for evaluating the performance of the proposed nano-coating system and reveal the differences in water ingress mechanisms which are specific to plain, superhydrophilic, overhydrophobic, and superhydrophobic specimens. The engineered nano-coatings were designed to improve hydrophobic response with inducing the required roughness pattern and introducing the photocatalytic performance. The effectiveness of the coatings was assessed using high-resolution neutron imaging (HR-NI), SEM, CLSM, and XRD techniques. High-resolution neutron imaging revealed that the superhydrophobic coating effectively prevents water ingress into the porous ceramic substrate, whereas water imbibition was observed for superhydrophilic coating during the test duration. The moisture transport kinetics was modeled based on the Richards equation for plain ceramic and superhydrophilic specimens using obtained penetration depth values from HR-NI. SEM, CLSM, and XRD studies confirm the desired TiO_2_-doped zinc phosphate coatings with increased surface roughness, photocatalytic reactivity, and chemical bonding. The research results demonstrated that a two-layer superhydrophobic system is capable of creating effective water barriers on the surface with contact angles of 153°, which remained effective even after surface damage.

## Introduction

Coatings play a significant role in protecting various materials from decay and deterioration^1^. Thin phosphate-based coatings that repel water, decompose organic and inorganic compounds, and provide anticorrosive properties are an innovative idea that combines crucial material protection potential and environmental benefits^2^.

**Figure 1:**
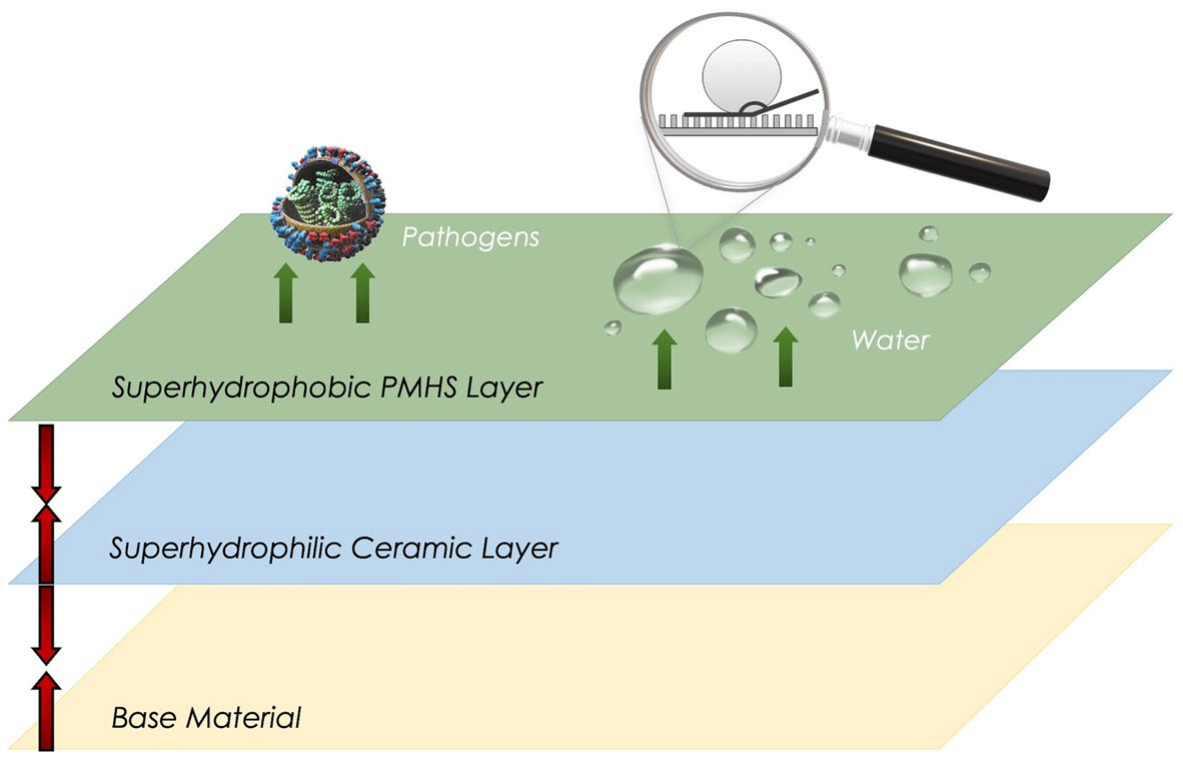
Schematic representation of the proposed coating system.

By combining the technologies of superhydrophilic phosphate-based ceramic cements, titania nanoparticle photocatalysts, and hydrophobic modification, we have developed a coating system for the treatment of concrete and other porous materials. Here, the base coat is an anticorrosive ceramic zinc phosphate thin film that has been doped with photocatalytic titanium dioxide (TiO_2_) nanoparticles tuned for self-cleaning and atmospheric remediation^3–5^. Previous studies have indicated that such TiO_2_ materials, when exposed to sunlight, can effectively oxidize the adsorbed pollutants and organic matter deposited on the surfaces, demonstrating self-cleaning and anti-graffiti properties^6–9^. It was also reported that car exhaust emissions, mainly NO_x_, but also a range of Volatile Organic Compounds (VOCs), can be chemically degraded on a TiO_2_ photocatalyst in the presence of UV light^6,7,10^. In wastewater treatment, the use of TiO_2_ has proved to be successful in the removal of harmful pollutants, including heavy metals^11,12^. This base coat, designed to induce surface roughness, is further treated with a siloxane-based compound to achieve an over- or even a super-hydrophobic response, which helps to repel and prevent the ingress of water and other corrosive elements. It was reported that hydrophobic coatings have potential applications in green engineering^13,14^. A schematic of the proposed coating system is presented in Fig. 1.

**Figure 2:**
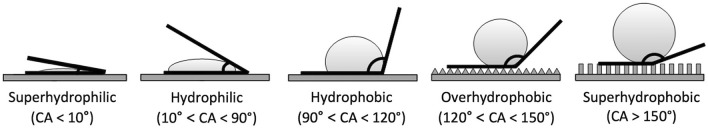
Various wetting states of the surface and corresponding water contact angles.

The wetting state of a surface can be described by the contact angle (CA) that water exerts when placed on the surface. Surfaces that are easily wetted by water are considered hydrophilic, with contact angles of less than 90°. If wetting occurs within one second and the contact angle is less than 10°, the surface is considered superhydrophilic^15^. Both hydrophilic and superhydrophilic surfaces, because they allow water spread, help to facilitate the photocatalytic reaction^16^. Surfaces that repel water have contact angles higher than 90° and are considered hydrophobic. This state can be further divided into overhydrophobic and superhydrophobic surfaces, which can be achieved by introducing hierarchical roughness^2^. Either nano- or micro-particles are needed to elevate the surface into the overhydrophobic state, and a combination of micro- and nano-roughness is necessary to achieve the superhydrophobic state. Different wetting states of the surface are presented in Fig. 2^17^.

Commonly, for CA and roll-off angle studies, a goniometer equipped with a high-speed camera and a tilting table is used^13,18,19^. In addition, as presented for this study, high-resolution neutron imaging provides a suitable method to determine CA. Simultaneously, thanks to the high neutron scattering cross section for hydrogen, it allows visualizing the water transport through the coating and base material^20,21^. The use of a neutron microscope detector was reported for the determination of water evaporation kinetics^22^ and the visualization of water transport in porous gas diffusion layers of fuel cells^23^.

Visualizing the absorption of water into uncoated and coated samples may be a useful technique for gaining a deeper understanding of this phenomenon. In addition to experimental methods, a speedy and cost-effective numerical simulation may be used for this task. For example, the moisture distribution in an unsaturated porous medium (e.g., soil) may be determined using Richards equation^24^. There are some analytical solutions that can be found in the literature as well^25^. Besides the analytical solutions, semi-analytical^26^, approximate^27^, empirical^28^ and even completely arbitrary methods^29^ have been developed over the past few decades. However, the complexity of the equation described by Gray and Hassanizadeh^30^ leads to further development of numerical solutions; some of these are presented in the literature^31–36^.

Four scenarios involving water droplets falling on the porous ceramic substrate were evaluated in this study: (1) the response of an untreated base material, a porous ceramic tile; (2) the response of a base material coated with a roughness-inducing layer of TiO_2_-doped zinc phosphate; (3) the response of a hydrophobically treated base material with a ceramic coating layer; and (4) the response of a mechanically scratched hydrophobic sample. For the present study, Richards equation for the sample specimen has been solved for the first two cases using a commercial software package, COMSOL Multiphysics^37^.

This reported experiment provides insights into how developed coatings change the wetting state of the surface of a porous substrate and how coating affects water transport. Based on the observations, two simulations, one for the reference (hydrophilic) surface case and one for the superhydrophilic coated surface case, were created for tracking the moisture transport in porous media. The employed models were based on Richards equation developed for modeling the unsaturated water–air flows in porous ceramics^24^.

## Methods

### Sample preparation

As a substrate, unglazed ceramic tiles were used. Each of the samples, prior to the coating application, was reduced to the desired size (approximately 7 × 7 × 5 mm cuboids), ultrasonically cleaned in water, and oven dried at 105 °C for two hours.

Hydrophilic and hydrophobic coating solutions were prepared. To prepare the hydrophilic base coat formulation, ZnO nanoparticles (supplied by Sigma Aldrich) were added to the solution of 85% phosphoric acid and water at a 1:10 stoichiometric ratio. The solution was mixed continuously on the hotplate for 15 min. Then, TiO_2_ nanoparticles (supplied by Sigma Aldrich) were added and mixed for an additional 15 min. The weight ratio between ZnO and TiO_2_ was 0.875, and the combined oxides to acid ratio was 1.057.

The hydrophobic coating solution was prepared by a gravimetrical addition of polymethylhydrogen siloxane oil (supplied by DOW chemicals) to the solvent (99% isopropanol) to obtain a 1 wt.% active ingredient solution. The solution was mixed vigorously over a period of 60 s.

The prepared coating solutions were applied onto the top side (7 × 7 mm) of previously prepared specimens by the spray deposition technique. The sequence of the application was as follows. First, the hydrophilic coat was applied, followed by one hour of oven heat treatment at 250 °C. The hydrophobic solution was applied by spraying cooled-to-room-temperature specimens, and further conditioning was performed at room temperature for 24 h.

### Methodology

**Figure 3:**
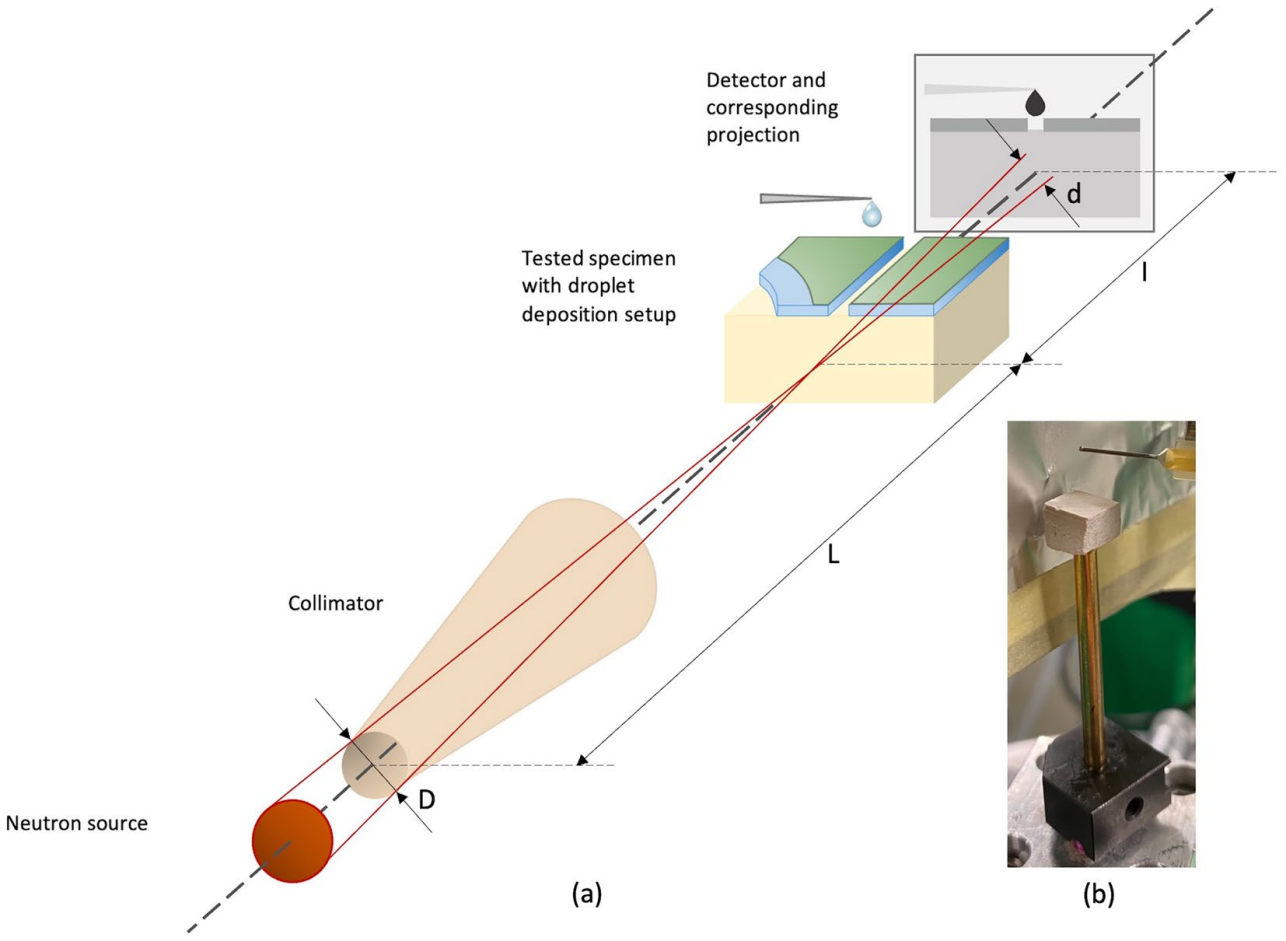
Neutron imaging setup: (**a**) schematic of experimental geometry of neutron imaging; and (**b**) experimental setup with sample on the holder and droplet deposition setup.

The morphology and structural characteristics of the samples were studied using a high-resolution JEOL JSM-6460L scanning electron microscope (SEM). A Denton Desk II Sputter Coater was used to sputter SEM specimens with a gold/palladium layer to improve the sample conductivity, reduce the charging of the surface, and increase the quality of the micrographs. Secondary electrons accelerated to 9 kV were used as a source. Every image was taken on 36 nm spot size. A LEXT OSL model-4100 confocal laser scanning microscope (CLSM) with an MPLFLN10 lens was used to characterize the surface roughness. An X-ray diffraction (XRD) analysis with Cu Ka radiation was performed using a Bruker AXS D8 Discover A25. The pore structure of the substrate material was characterized by a mercury intrusion porosimeter (MIP). The Micrometrics AutoPore IV 9500 porosimeter with a maximum pressure of 60,000 psia, a pressure accuracy of 0.1 psia, and a solid sample penetrometer with a stem volume of 1.131 cm^3^ was used for the test. High-resolution neutron imaging (HR-NI) was performed using PSI Neutron Microscope detector^38^ at the ICON cold neutron imaging beamline^39^ at the Swiss spallation neutron source, SINQ^40^. The detector was placed at a distance L of 9.45 m downstream from the beam-defining circular aperture of diameter D equal to 40 mm, thus leading to an L/D ratio of approximately 236. A schematic of the neutron imaging geometry is visible in Fig. 3a^41^. The mean distance between the sample and the detector was equal to 4.5 mm. The detector was equipped with a 3.5 µm highly isotopically enriched 157-gadolinium oxysulfide scintillator screen^38,42^.

### Experiment

#### High-resolution neutron imaging

For the high-resolution neutron imaging (HR-NI) investigations, the ceramic cuboid specimens were glued to the sample holder, Fig. 3b, that was positioned on the sample stage of the neutron microscope detector. Water droplets (5 µL) were delivered using a needle remotely operated titration system^43^.

After a sample was successfully positioned in the range of the scintillator, images of the open background, dark current, and sample projections at various wetting stages were taken. The Fiji ImageJ software^44^ was used to obtain final, refined projections by utilizing the Beer-Lambert (B-L) law, Eq. (1), principles that relate the attenuation of a neutron beam with an exponential law of a material effective thickness (x), and the linear attenuation coefficient of the investigated material (∑)^45^.

1 $$I={I}_{0}{e}^{-\sum \mathrm{x}}$$

From the B-L law, it is known that *T*, the transmittance (the computed final image), is represented by the intensity of the neutron beam transmitted through the sample (*I*), divided by the incident intensity (*I*_0_), which corresponds to the projection image (*I*_P_) divided by the open beam image (*I*_*OB*_). Both parameters were adjusted by subtracting the dark current image (*I*_*DC*_) to enhance the quality of projection^46,47^; thus, Eq. (2) was used to generate the final images:

2 $$T=\frac{I}{{I}_{0}}=\frac{{I}_{P}-{I}_{DC}}{{I}_{OB}-{I}_{DC}}$$

#### Simulation and modeling

Solving Richards equation yields the saturation distribution in granular materials such as soils in the “vadose zone,” also known as the unsaturated zone situated immediately above the water table. The following equation is used to calculate the soil’s saturation of porous material; the derivation details for Eq. (3) are available in reference^48^.

3 $$\epsilon \frac{\partial S}{\partial t}=\nabla \cdot {k}_{r}\frac{K}{\mu }\left(-\frac{d\langle {p}_{C}\rangle }{dS}\right)\nabla S+\frac{K\rho g}{\mu }\left(\frac{d{k}_{r}}{dS}\right)\frac{\partial S}{\partial z}$$

where *ϵ* is the porosity of the material (ceramic), *S* is the saturation of the liquid (water), and *k*_*r*_ is the relative permeability of the wetting phase (i.e., water). Since it is the tendency of a liquid to wet the porous media, *K* is the permeability of the porous media (ceramic tile), *p*_*c*_ is the capillary pressure, *g* is the acceleration due to gravity, and ρ is the density of the ceramic tile. Saturation is equal to the ratio of liquid volume to the pore volume in a representative elementary volume of a porous medium. It is a dimensionless quantity that lies between S = 0 (completely dry porous medium) and S = 1 (fully wet porous medium). Here, both the relative permeability and the capillary pressure can be expressed as separate functions of saturation, and hence Eq. (3) transforms to:

4 $$\epsilon \frac{\partial S}{\partial t}=\nabla \cdot \frac{0.04 K}{\mu }\frac{ {S}^{1.75}}{1-S} \nabla S+\frac{K\rho g}{\mu }3{S}^{2}\frac{\partial S}{\partial z}$$

In order to solve Eq. (4) numerically, we needed to determine the value for *K* using MIP, as discussed in the result section^49^.

In our experiment, the dry ceramic sample is regarded as an unsaturated zone. The saturation is set to unity at the upper surface of the ceramic block as soon as the water droplet touches it. The bottom wall’s boundary condition is set to zero, while the side walls’ boundary conditions are set to zero flux. These boundary conditions are listed in Table 1. This investigation’s simulations were performed using COMSOL Multiphysics, and Richards equation was solved using a finite element method with a six-noded triangular fine mesh.

**Table 1:** Boundary conditions for 3D simulation (see Fig. 4a for the coordinate system used for the simulation).

	Top surface (circular area)	Bottom surface	XZ planes	YZ planes
Saturation	*S* = 1	*S* = 0	$\frac{\partial S}{\partial y}=0$	$\frac{\partial S}{\partial x}=0$

## Results

### Material characterization

Different methods, such as scanning electron microscopy (SEM), X-ray powder diffraction (XRD), confocal laser scanning microscope (CLSM), and mercury intrusion porosimetry (MIP) were used to characterize the substrate and obtained coatings.

#### SEM observations

**Figure 4:**
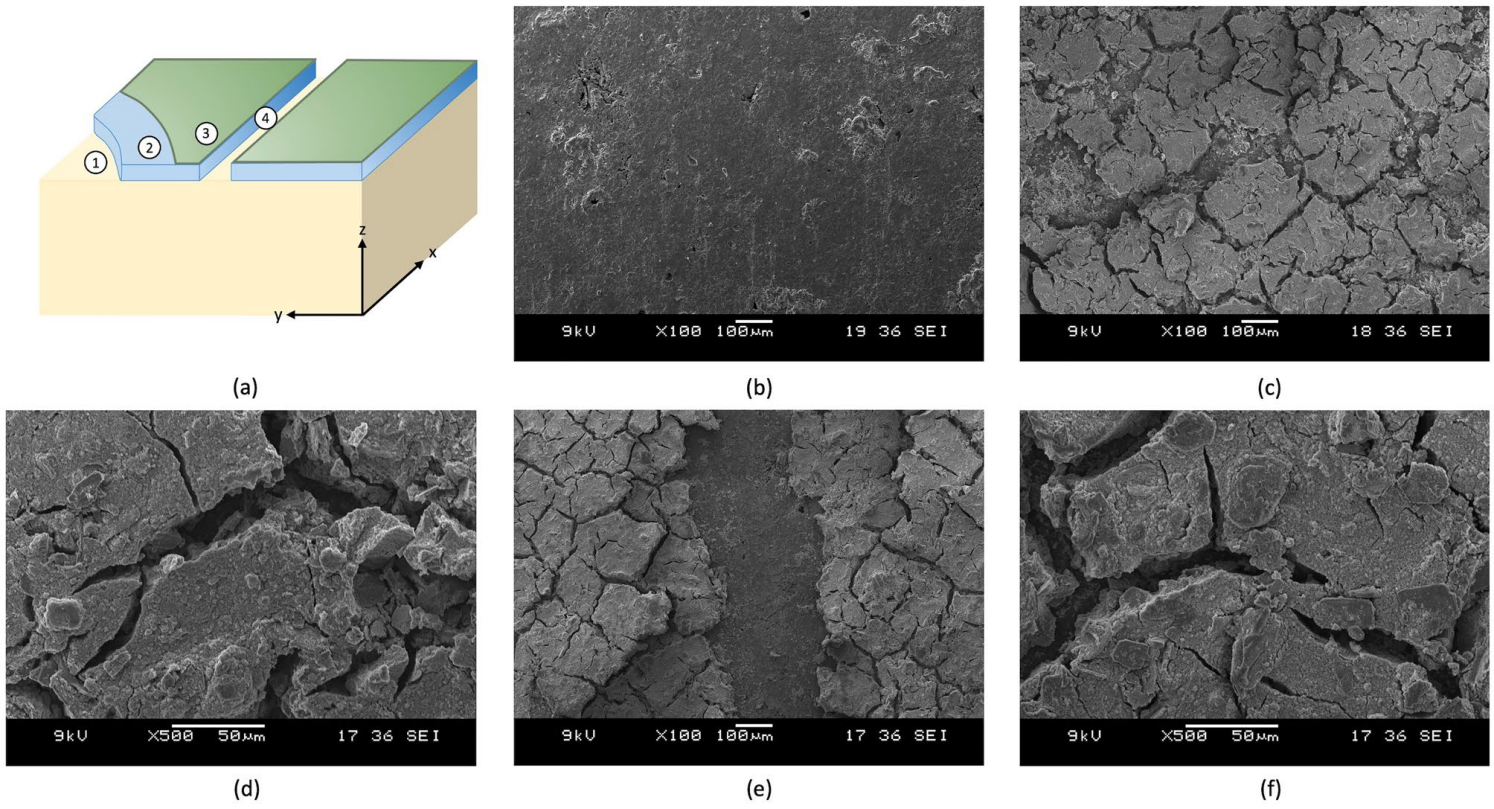
Schematic of measurement positioning and SEM micrographs: (**a**) the schematic of coating systems with the indication of the measurement positions; (**b**) the reference base material, position 1; (**c**) and (**d**) the superhydrophilic coating, position 2; (**e**) the single-scratched superhydrophobic coating, position 4; and (**f**) the superhydrophobic coating, position 3.

The morphology of the base material (AR) and the coating systems (X21 and X21H) is depicted in Fig. 4. A schematic of the coating layers and the corresponding locations of the measurement are described in Fig. 4a. The surface of the reference material that serves as a base for the coating is presented in Fig. 4b, with a visible pore structure. The surface treatment with the coating formulation systematically filled the pores and created the desired roughness at the surface of the specimen. The coating layer was composed of sharp edge crystals that suffered from hydrothermal cracks, which further enhanced the roughness and created conditions that favored obtaining high water contact angles^2^. There is no distinct difference between the hydrophilic surface visible in Fig. 4c,d, and the hydrophobic surface treatment depicted in Fig. 4e,f, which suggests that the hydrophobic layer is very thin. Figure 4e visualizes a scenario where a two-layer composite coating was manually scratched (with a scratch width of roughly 200 μm), and all layers were removed up to the substrate.

#### XRD investigation

**Figure 5:**
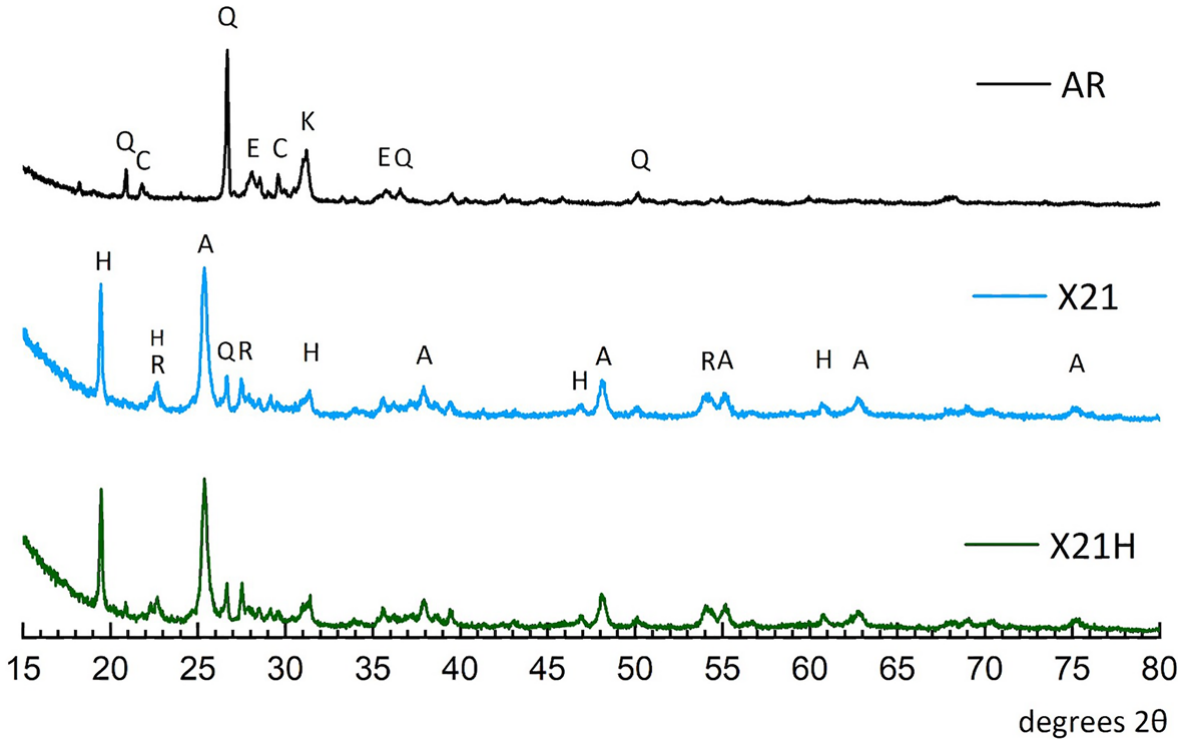
X-ray powder diffractogram of reference ceramic (AR), superhydrophilic coating (X21), and superhydrophobic coating (X21H).

The phase purity and crystallinity of the samples were examined using XRD; see Fig. 5. The reference sample (AR) was determined to be composed of quartz (SiO_2_, marked as Q), cordierite (MgFe silicate, marked as C), akermanite (CaMg silicate, marked as K), and enstatite (MgSiO_3_, marked as E). Peak occurrences matched the standard values from the Joint Committee on Powder Diffraction Standards (JCPDS) database. All XRD patterns reflect the existence of pure peaks; upon coating treatment, all base material peaks, except quartz, which was significantly reduced in intensity, were masked with the developed coating. New peaks emerged in the composite architecture, evidencing the presence of zinc phosphate (hopeite, Zn_3_(PO_4_)_2_·4H_2_O, marked as H), anatase (TiO_2_, marked as A), and rutile (TiO_2_, marked as R). When comparing X21 (superhydrophilic) and X21H (superhydrophobic) diffractograms, it can be noticed that there is no distinct difference in the composition. Based on that finding, conclusions about a very thin hydrophobic layer can be drawn, which correspond to SEM micrographs, where a hydrophobic topcoat cannot be seen. The composition balance using the Rietveld function from JADE software indicates that the coating is composed of 50.7% of anatase, 29.5% of hopeite, and 9.6% of rutile. The amount of 3.4% of the signal corresponds to quartz protruding from the surface. It was also determined that 6.8% of the sample was amorphous.

#### CLSM investigation

**Figure 6:**
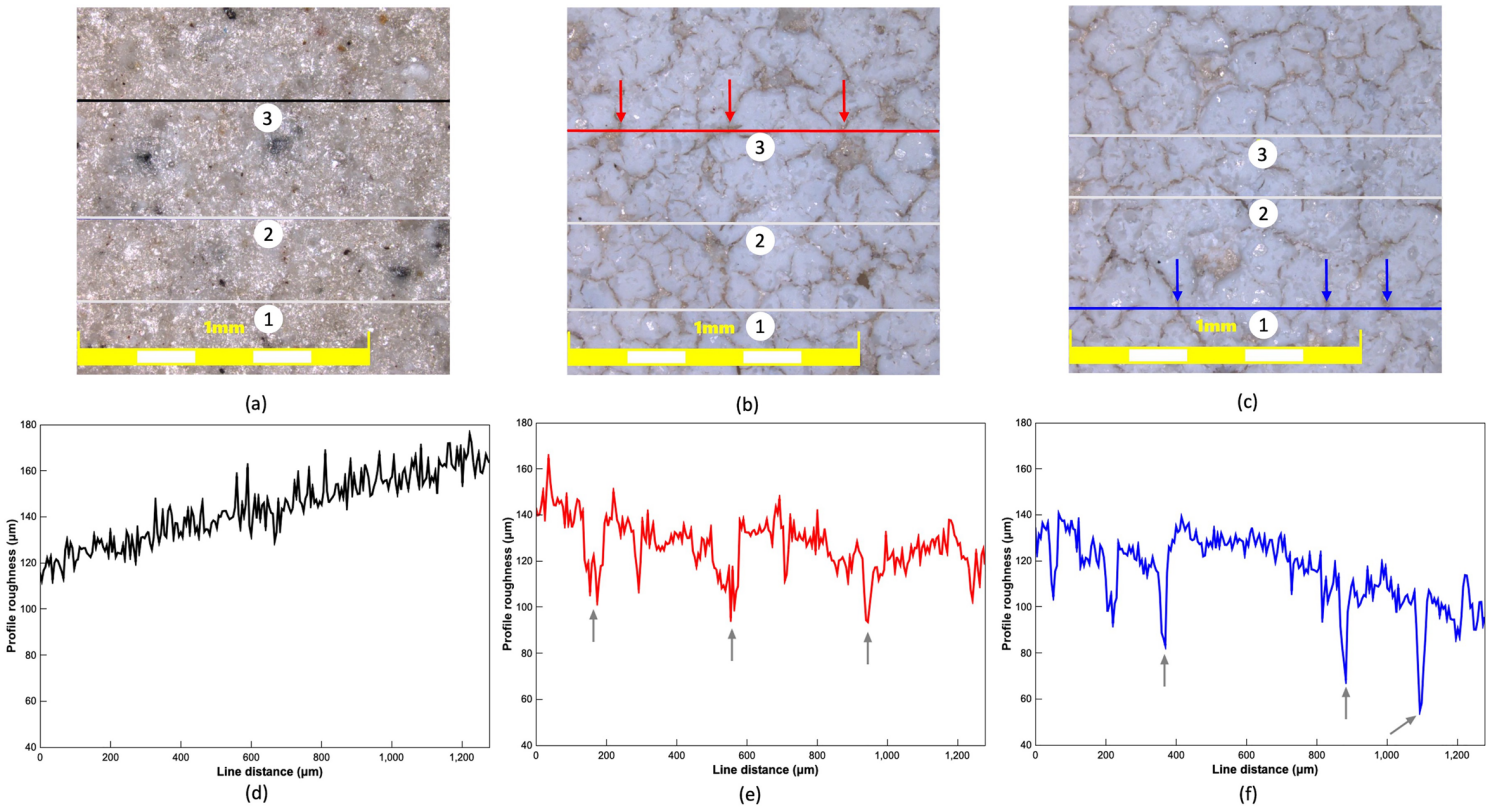
Confocal images with line roughness measurement positions marked: (**a**) the reference ceramic; (**b**) the superhydrophilic coating; (**c**) the superhydrophobic coating; and (**d**) through (**f**) line 3 profile roughness histogram for reference ceramic, line 3 for superhydrophilic, and line 1 for superhydrophobic coating, respectively.

A CLSM was used to characterize the roughness of the samples. Confocal images were taken with an XYZ step scanning mode at 10X magnification. Line roughness measurements were taken for each sample in three different positions. The obtained images and the measurement positions are marked in Fig. 6a–c. Profile roughness histograms for single line measurement were plotted in Fig. 6d–f, and the average roughness (Ra) parameter was determined and presented in Table 2.

**Table 2:** Roughness parameter (Ra) for reference ceramic specimen, superhydrophilic coating, and superhydrophobic coating.

Ra roughness	Reference ceramic	Superhydrophilic coating	Superhydrophobic coating
Line 1, μm	4.077	5.814	8.730
Line 2, μm	3.832	7.522	7.854
Line 3, μm	4.098	7.808	6.768
Average of three measurements, μm	4.002	7.048	7.784
Standard deviation, μm	0.148	1.078	0.983

By comparing the uncoated reference surface, Fig. 6a, with the coated surfaces, Fig. 6b,c, it is visible that the coating is not uniform, with distinct hydrothermal cracks reaching to the base material present (confirming the observations by SEM, Fig. 4). The width of the average hydrothermal crack was determined to be 0.7 μm using Fiji ImageJ software. The depth of three cracks at each specimen’s three measurement lines was measured, and the average thickness of the coating system was determined to be 37.3 ± 2.8 μm for the superhydrophilic specimen and 42.3 ± 3.4 μm for the superhydrophobic specimen. Locations of depth measurements were marked with arrows in Fig. 6b,c as compared with corresponding positions at the histograms in Fig. 6e,f.

It can be observed in Table 2 that the roughness increases with every applied coat. Application of the superhydrophilic coating increases the roughness approximately by 75% (from 4.0 to 7.0 μm) and further application of the hydrophobic treatment increases the roughness by an additional 10% (from 7.0 to 7.8 μm).

#### MIP study

The reference ceramic substrate material was evaluated using MIP to determine its porosity and permeability constants, the properties that were needed for creating the 2D and 3D simulations. Mercury with a surface tension of 0.485 N/m, a contact angle of 130°, and a density of 13.534 g/cm^3^ was forced through the pores of the ceramic at low and high pressures. While the low-pressure mode is sufficient to force mercury into large pores, high pressure is necessary to fill the small pores. Washburn equation, presented as Eq. (5), was used to determine the pore size (*D*_*P*_), which is inversely proportional to the applied pressure (*P*), the contact angle of mercury (θ), and its surface tension (γ), and has an influence on the obtained result.

5 $${D}_{p}=-\frac{4 \gamma cos\theta }{P}$$

**Figure 7:**
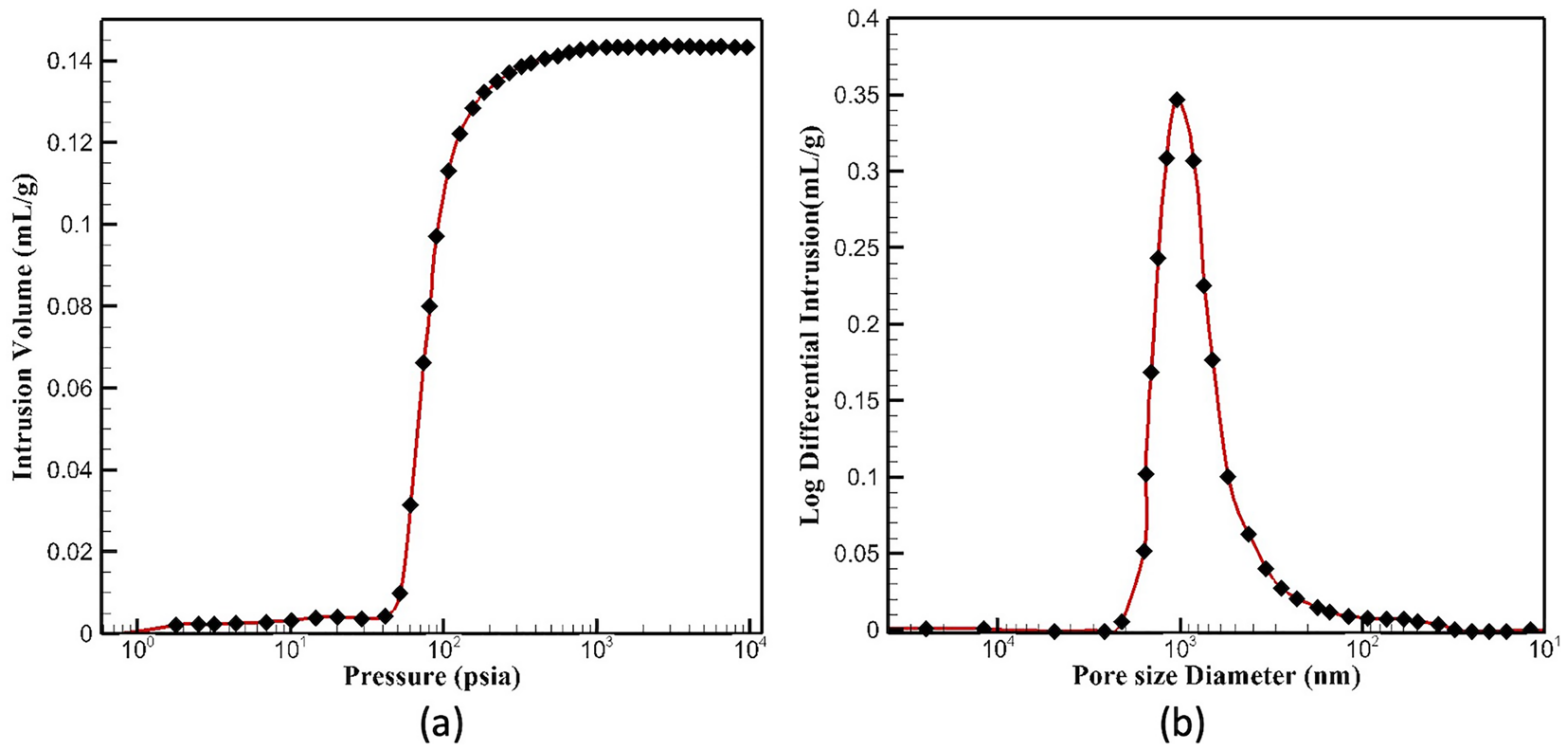
The mercury intrusion volume in the MIP test as a function of (**a**) pressure; and (**b**) pore size diameter.

The mercury intrusion volume is presented as a function of the pressure in Fig. 7a and as a function of the pore size diameter in Fig. 7b. The sample was determined to have 36% porosity and an average pore size diameter of 647.3 nm, with evenly distributed pores. The parameters presented in Table 3 were used for modeling. Detailed calculations for the MIP can be found in reference^49^. Besides MIP, two additional methods, the falling head permeameter and the SEM micrograph analysis, were used to determine the permeability as well as the porosity of the specimen. All three methods provided a good estimation of permeability as required for the simulation of water transport.

**Table 3:** Parameters calculated based on the MIP experiment.

Calculated parameter	Value
Porosity, ε	0.36
Permeability constant, *K*	3.86 × 10^–16^ m^2^
Average pore diameter, *D*_*P*_	647.3 nm
Bulk density at 0.53 psia, ρ	2.53 g/cm^3^

### Wettability of surfaces

The outcomes of the neutron microscope imaging and simulation of moisture movement are presented in this section. Each sample was positioned in the sample holder, as visible in Fig. 3b, and a neutron beam was traveling across the x-axis of the sample, as depicted in Fig. 4a.

#### Response of reference ceramic material and superhydrophilic coating

**Figure 8:**
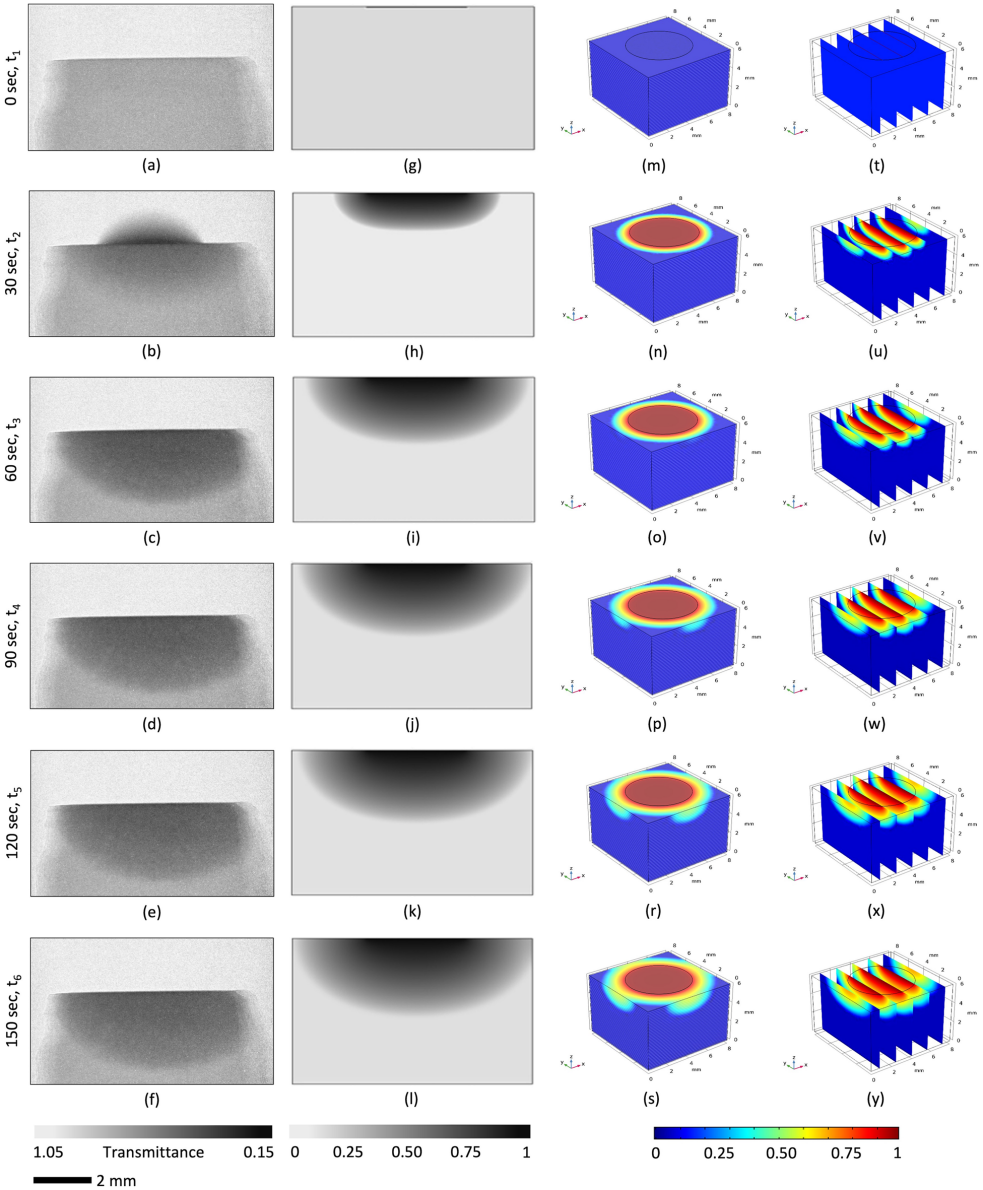
Analysis of moisture imbibition through the hydrophilic, untreated porous ceramic material: (**a**) through (**f**) experimental results; (**g**) through (**l**) corresponding 2D simulation results; and (**m**) through (**y**) the visualization of moisture infusion into a 3D ceramic block for 6-time steps (t_6_ > t_5_ > t_4_ > t_3_ > t_2_ > t_1_).

**Figure 9:**
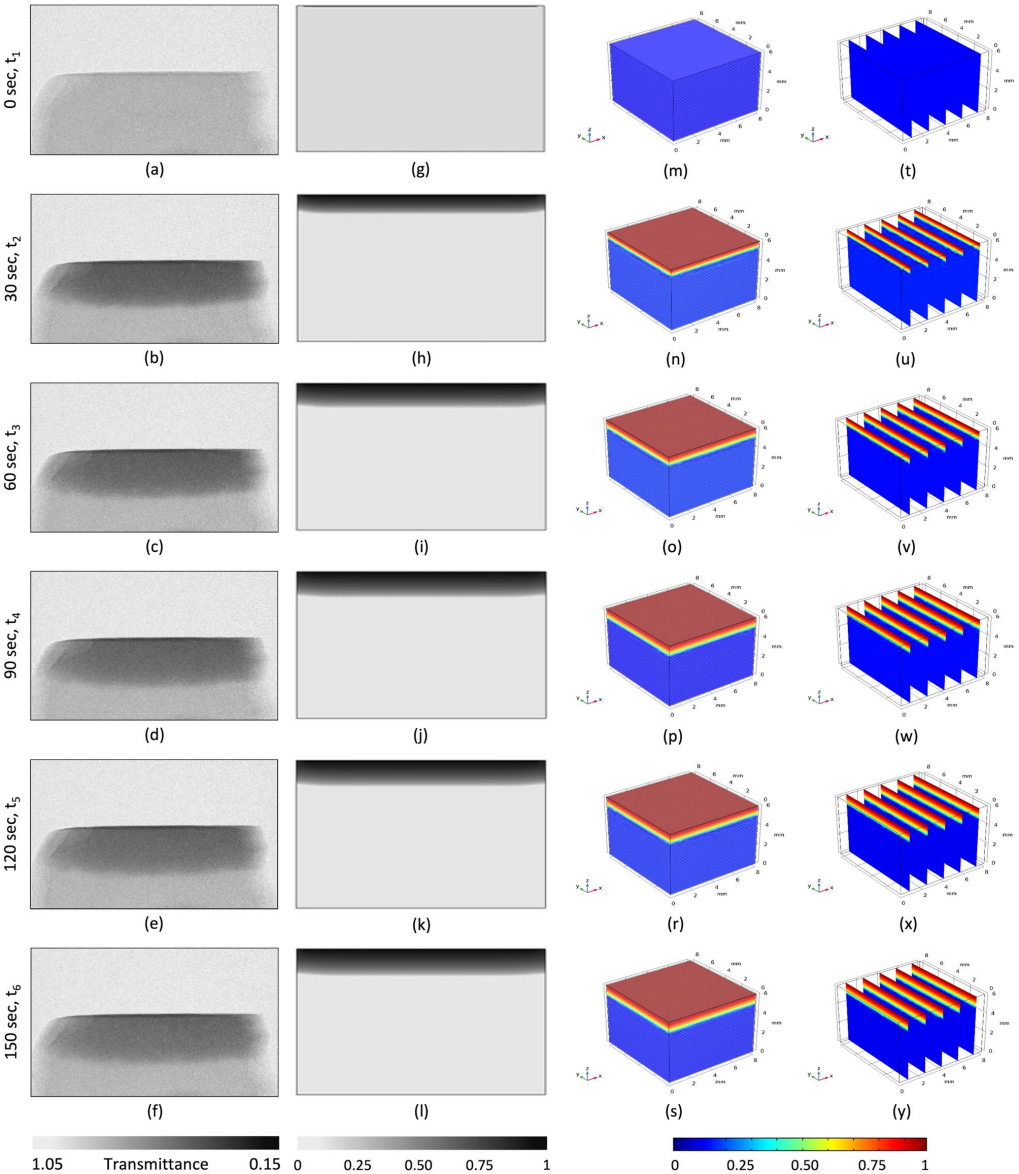
Analysis of moisture imbibition through the superhydrophilicaly-treated porous ceramic material: (**a**) through (**f**) experimental results; (**g**) through (**l**) corresponding 2D simulation results; and (**m**) through (**y**) the visualization of moisture infusion into a 3D ceramic block for 6-time steps (t_6_ > t_5_ > t_4_ > t_3_ > t_2_ > t_1_).

An application of the first coat enhances the reference material hydrophilicity and creates a superhydrophilic surface. A water droplet placed at the top of the specimen immediately soaks into the substrate and is distributed evenly within the volume of the block. This is perfectly visible on the obtained neutron images, Fig. 8a–f and Fig. 9a–f. Each image was additionally modeled to create 2D and 3D simulations, as seen in Fig. 8g–y and Fig. 9g–y.

According to the findings shown in Fig. 8a–f, the level of water saturation in the ceramic is equal to zero (*S* = 0) prior to the appearance of water droplets on the surface of the sample. Each experimental neutron image corresponds to 30 s of measurement. The whole domain in Fig. 8g is shown to be grey, and it can be deduced that there is no moisture present in the domain. Figure 8b demonstrates that the water droplet just lands on the top surface of the ceramic tile, and the area beneath the droplet is completely black. Furthermore, the color gradually fades towards the bottom, which indicates that the saturation gradually decreases in that direction. This is also visible in the result of the simulation reported in Fig. 8h. After 1 min, the top surface of the tile has no water on it, which indicates that the water droplet has completely infused into the sample, Fig. 8c. Similarly, the saturation zone spreads over a larger area, as seen in Fig. 8c. The saturation front continues to progress over time, as depicted in Fig. 8j–l and Fig. 8d–f. The image proves that over a period of 90 s, the saturation front moves a little distance closer to the bottom; see Fig. 8d–j. After enough time has passed, the progression of the saturation front will no longer be detectable. This is because there is no longer any supply of water droplets that are deposited onto the surface, as evident from Fig. 8e,f, and Fig. 8k,l. It is quite clear that the simulation can qualitatively replicate the experimental observations with a large degree of accuracy (the small divergence as observed can be due to local heterogeneity in material properties and deviations in the dimensions of the ceramic sample).

**Figure 10:**
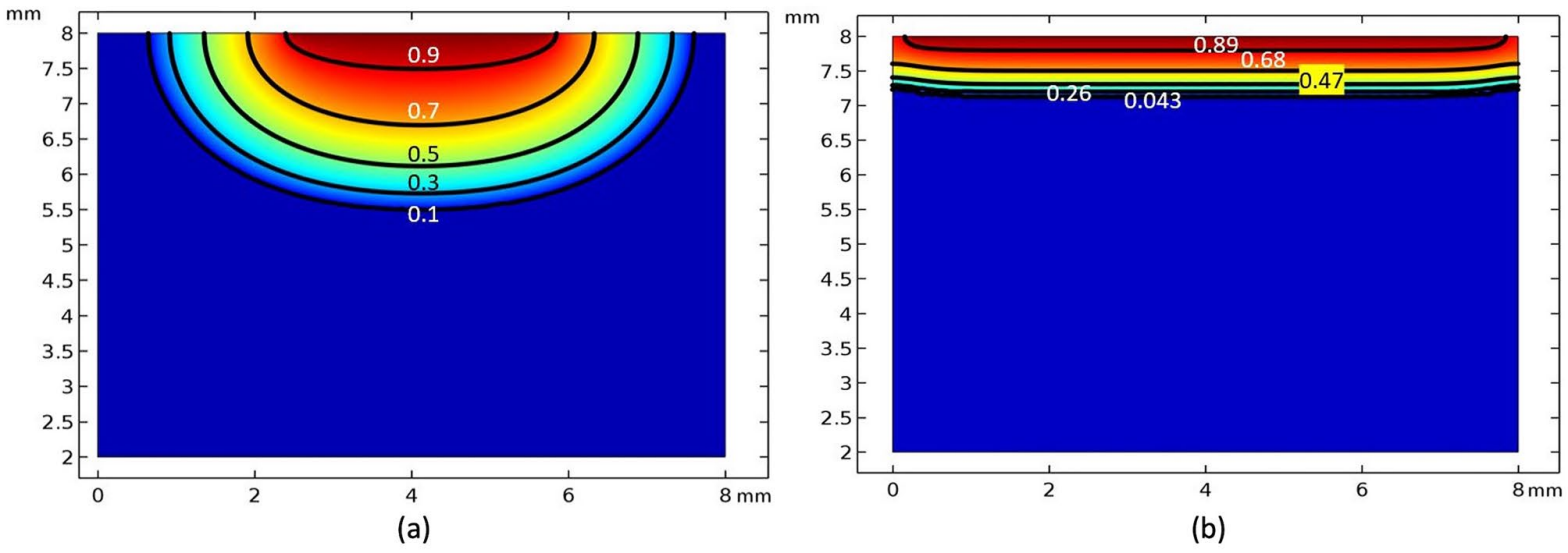
The saturation level predicted by the simulation after 50 s: (**a**) without coating; and (**b**) with superhydrophilic coating.

Figure 9a–f presents the experimental results of water being absorbed by a porous ceramic tile with a superhydrophilic coating, and Fig. 9g–l represents the corresponding 2D simulation. In the simulation, it is assumed that the water droplet rapidly spreads all over the top surface and completely wets the top surface very quickly so the boundary condition corresponding to the top surface was set as S = 1 at the start of the simulation, and then maintained throughout the simulation. Each experimental neutron image corresponds to 30 s of measurement. It is visible that, until a drop of water settles on the ceramic tile, the surface color remains unchanged at a uniform gray, Fig. 9a–g. As soon as the water droplet contacts the ceramic tile, a surface phenomenon takes place, known as the droplet spreading out over the surface of the specimen. As mentioned earlier, dry ceramic material initially has a saturation of zero, but after a drop of water spreads over its surface, it reaches a saturation of one. The iso-saturation lines are flat and parallel to the top surface; the gradient in saturation is downwards. However, the depth of penetration is much shallower here than it was before. Also, the saturation front is straight compared with the half-elliptical front for the reference sample without coating. After 50 s, the saturation contour plot in Fig. 10b can be compared with the saturation contour plot distributed in Fig. 10a for an uncoated sample. It is evident from Fig. 10 that the saturation front for the sample with the hydrophilic coating, Fig. 10b, is linear and has a shallower penetration depth, while the saturation front for the sample without the coating is elliptical and has a greater depth of penetration, as depicted in Fig. 10a.

A 3D simulation was conducted to study the observed phenomena more realistically. After creating a sample with unit dimensions and adding the material attributes corresponding to the sample in COMSOL, the next step was to solve Richards equation for the 3D geometry using the boundary conditions (Table 1). Figure 8 (right-hand side) provides a 3D visualization of the progressive imbibition that occurs in the reference porous ceramic blocks that do not have any coatings. To facilitate an understanding and visualization, the ceramic block was cut into five cross sections along the yz plane, Fig. 8m–s, and 40 cross sections along the xy plane, Fig. 8t–y. The beginning of the experiment suggests that the sample’s top surface (the circular region) has a saturation level that is very close to one. This can also be observed in Fig. 8u. In the xy planes, it does so with a smooth progression in the radial direction, whereas in the yz planes, it does so with a half-elliptical saturation front. The penetration is at its greatest point directly at the center of the plane, and it progressively shallows out as it moves outward. In addition, the saturation level is at its highest along the xy plane at the surface closest to the top. From there, it progressively decreases as it moves closer to the edge, and then it changes to the next plane. As time passes, there is a general trend toward progressively reducing the saturation levels across the board. Similarly, Fig. 9m–s and Fig. 9t–y depict the 3D infusion of moisture in a single layer-coated sample. It is evident from Fig. 9u that, for this case, there is a surface phenomenon by means of which the fallen droplet spreads rapidly on the top surface of the specimen. After that, the saturation front starts to propagate uniformly in almost a 1D manner towards the bottom.

#### Superhydrophobic response

**Figure 11:**
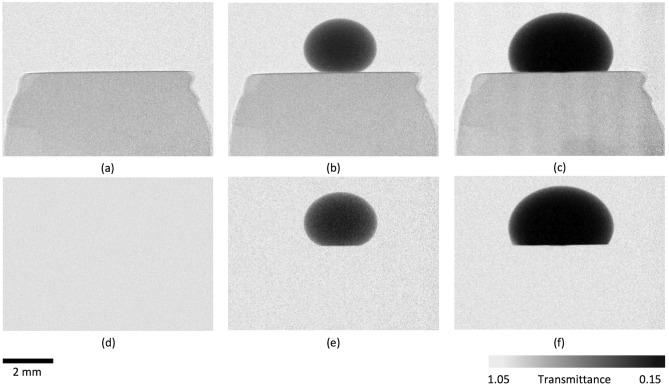
Neutron images of the hydrophobic sample: (**a**) and (**d**) represent scenarios before water droplets reach the surface; (**b**) and (**e**) after one droplet reaches the surface; and (**c**) and (**f**) represent the scratched sample with two droplets atop.

The addition of the siloxane treatment switches the wettability of the surface from superhydrophilic to superhydrophobic. Obtained neutron projections are depicted in Fig. 11 and correspond to Cassie-Baxter’s state^50,51^. It is visible that the water droplet (dark) forms a sphere at the surface of the specimen. It is worth mentioning that Fig. 11a–d, as well as Fig. 11b–e and Fig. 11c–f, are the same projections presented differently. The projection image of the static state, before any droplet reaches the surface, was subtracted in the Fiji ImageJ software from projections depicted in Fig. 11a–c, to give dynamic projections of Fig. 11d–f, respectively. This approach allows tracking any potential moisture ingress into the coating and the substrate material.

**Figure 12:**
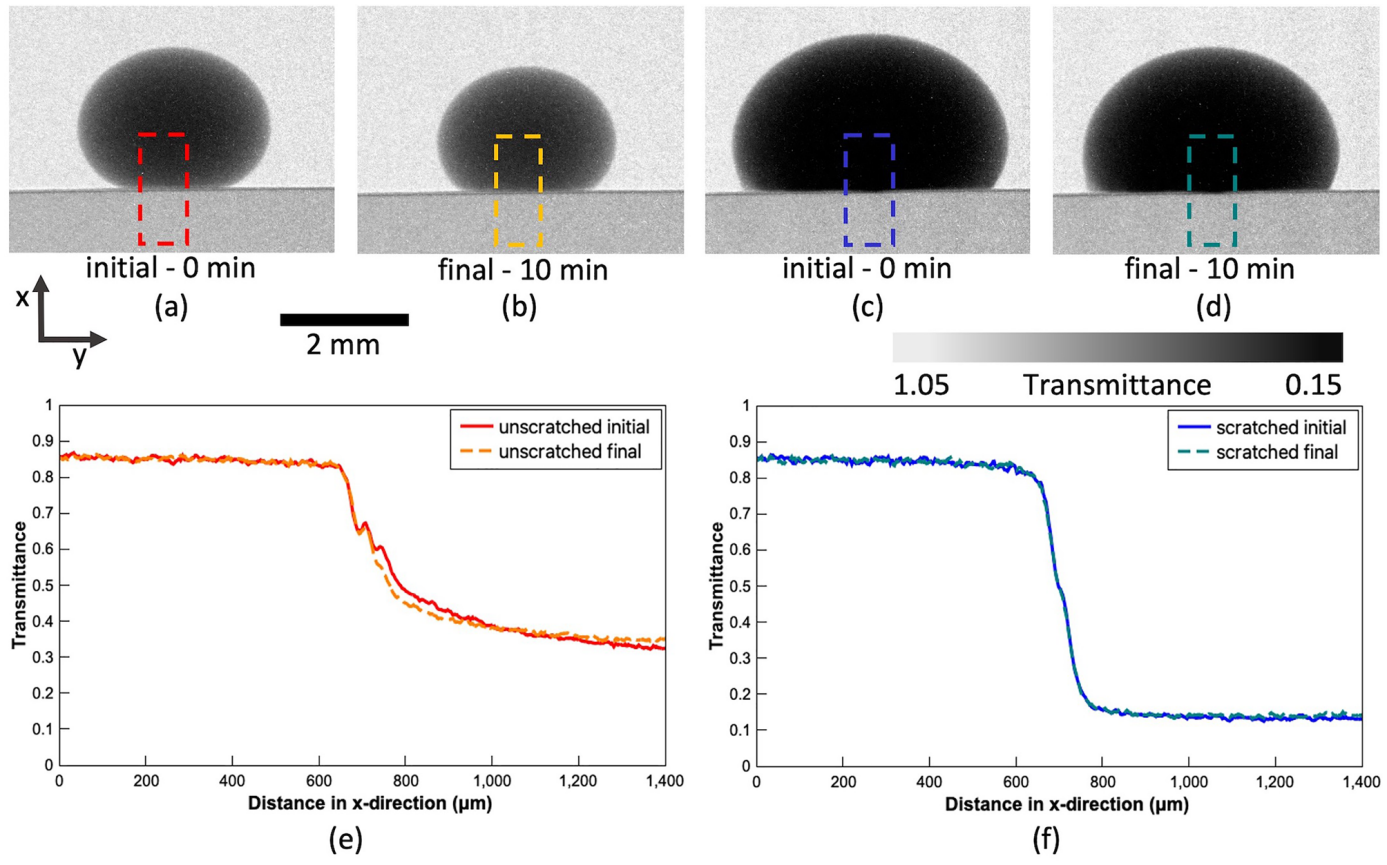
Neutron images of the unscratched and scratched hydrophobic sample with the gray value plot measurement: (**a**) the initial measurement for unscratched sample; (**b**) the final measurement for unscratched sample after 10 min; (**c**) the initial measurement for scratched sample; (**d**) final measurement for scratched sample after 10 min; and (**e**) and (**f**) are the gray value plots corresponding to (**a**) through (**b**) and (**c**) through (**d**) images, respectively.

The transmittance value can be used to evaluate whether water perforates the coating and travels through the substrate pores. The higher the value, the brighter the pixels. Thus, water that attenuates neutrons will impose the reduction of pixel brightness. The measurement area for the unscratched hydrophobic sample, upon which transmittance plots were created, is presented in Fig. 12a–b. Similarly, for the hydrophobic sample with imposed coating discontinuation, it is presented in Fig. 12c–d. The top of each selection rectangle is the initial point of the measurement (distance in x direction corresponding to 0 μm), and the bottom of the selection rectangle is the final point of the measurement area (distance in x direction corresponding to 1400 μm). The distance where transmittance readings change from values corresponding to the droplet into the values corresponding to the substrate is in the middle of the graph between 650 to 750 μm. The axes indicator was embedded into the figure to help to understand the graph-image correlation. Transmittance plots for two scenarios are shown in Fig. 12e–f. The area of the specimen has transmittance values around 0.85. Any water uptake would reduce this value, and, as visible in the graphs, there is no change between the initial (red and blue) and final (orange and green) curves. It is visible that even with a distinct scratch, as seen in Fig. 4d micrograph, the coating prevents water from penetration (no water visible in the substrate and no change in transmittance value).

#### Contact angle evaluation based on neutron images

**Figure 13:**
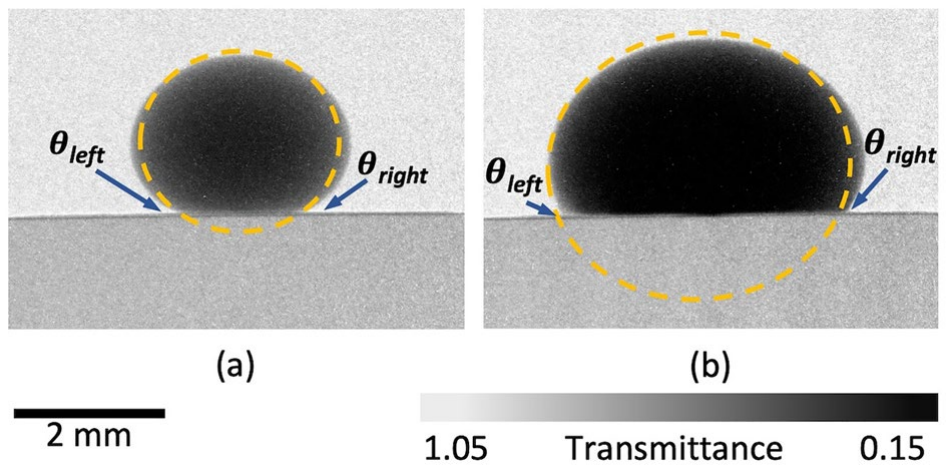
Water contact angle measurements: (**a**) the unscratched sample; and (**b**) the scratched sample.

The water contact angle was measured using the Fiji ImageJ contact angle plugin, according to the procedure developed by Brugnara^52,53^. First, images were magnified; then, two base points were selected at the positions where the droplet edges touched the surface. Further, three additional points were chosen along the drop profile. Next, the program performed the best-fit analysis according to the ellipse and circle fitting and determined theta angles that could be used for the calculation of a contact angle. Figure 13a,b present images with model fittings for the unscratched and scratched sample, respectively. The position of the two measured thetas is presented in Fig. 13b.

For the unscratched scenario, Fig. 13a, theta right (θ_*right*_) and theta left (θ_*left*_) were determined to be 27.5° and 27.4°, respectively. The contact angle can be calculated by subtracting the measured theta value from 180°; thus, the obtained contact angles are 152.6° and 152.5°, for the right and left theta, respectively. For the unscratched sample, the attained contact angles exceeded 150°, which situates the wetting state of the surface in the range of superhydrophobicity^17^. For the scratched scenario, Fig. 13b, the contact angle was calculated to be 118° and 121.2°, which situates the surface at the verge of overhydrophobicity^7^. The difference between the right and left measurement is likely because the droplet sitting atop the scratch will not be centrally symmetrical, even though, due to mechanical interference with the coating’s continuity, the surface still strongly repels water and prevents any moisture ingress, as obtained via the gray value measurement, Fig. 12f. Each measurement was repeated three times, and the uncertainty of the measurement was ± 3°. The results obtained are presented in Table 4.

**Table 4:** Contact angle measurement values for unscratched and scratched hydrophobic samples.

Parameter,°	θ_*C*_	θ_*E*_	θ_*left*_	θ_*right*_	CA_*left*_	CA_*right*_	CA_*average*_
Unscratched hydrophobic	34	27.4	27.4	27.5	152.6	152.5	152.6
Scratched hydrophobic	69.1	60.9	62	59.8	118.0	121.2	119.6

## Conclusions

The reported research confirmed the feasibility of a TiO_2_-doped zinc phosphate compound: (1) to introduce a coating system with desired roughness characteristics capable of enhancing the hydrophobic response of the material; (2) to introduce a system with photocatalytic reactivity, aiding decomposition of harmful to environment species; and (3) to create a chemical bond between layers of the nano-coatings and the substrate.

A neutron imaging technique demonstrated a successful water ingress blockade for coatings developed with the proposed approach. The created superhydrophobic surfaces had contact angles of about 153° and even scratched surfaces (scratch width of roughly 200 μm) reached the overhydrophobic properties with contact angles of about 120°.

High-resolution neutron imaging revealed that the application of a superhydrophobic coating prevented water ingress into the sample during the timespan of the test (10 min). It was observed that water neither ingressed into the coating nor through the coating into the porous ceramic substrate.

SEM and CLSM studies indicated the coating morphology and visualized the roughness of the coating. Additionally, CLSM was used to determine the thickness and roughness of the proposed coating system. It was found that the application of the proposed hydrophilic coat increases the roughness of the ceramic substrate by 75%, from roughly 4.0 μm to 7.0 μm, and by 95% when treated with a complete coating system, from 4.0 μm to 7.8 μm. The thickness of the hydrophilic coating was determined to be 37.3 ± 2.8 μm, while the application of a hydrophobic coat increased the overall thickness to 42.3 ± 3.4 μm. XRD was used to determine the substrates and the coatings’ chemistry and confirmed the formation of the envisioned zinc phosphate layer.

It was found that the proposed superhydrophilic surface treatment can help to evenly distribute water over the surface for uniform transport through a treated interface and allow uniform water seepage through the pores. Models based on the experimental results were drawn and correlated well with the observations. In contrast, for a sample without Zn-phosphate, TiO_2_ doped coating, the saturation front was elliptical, reaching greater penetration depth, as opposed to the hydrophilic coating’s response with a linear saturation front characterized by reduced penetration depth.

In the future, proposed coatings can also be tested to determine the roll-off angle behavior and dynamic interaction of droplets of different chemistry (i.e., salt solutions). Further research and modeling are needed to fully understand and visualize the movement of water over a longer period of time under dynamic conditions imitating a continuous flow over the surface.

## Supplementary Information

Supplementary Information.

## Data Availability

Data are available upon request; high-resolution neutron imaging and CLSM raw data are provided.
